# Corticospinal excitability measurements using transcranial magnetic stimulation are valid with intramuscular electromyography

**DOI:** 10.1371/journal.pone.0172152

**Published:** 2017-02-23

**Authors:** Rebekah L. S. Summers, Mo Chen, Teresa J. Kimberley

**Affiliations:** 1 Program in Physical Therapy, Department of Physical Medicine and Rehabilitation, University of Minnesota, Minneapolis, MN, United States of America; 2 Department of Physical Medicine and Rehabilitation, Rehabilitation Science, University of Minnesota, Minneapolis, MN, United States of America; 3 Institute for Engineering in Medicine, University of Minnesota, Minneapolis, MN, United States of America; Nanjing Normal University, CHINA

## Abstract

**Objectives:**

Muscular targets that are deep or inaccessible to surface electromyography (sEMG) require intrinsic recording using fine-wire electromyography (fEMG). It is unknown if fEMG validly record cortically evoked muscle responses compared to sEMG. The purpose of this investigation was to establish the validity and agreement of fEMG compared to sEMG to quantify typical transcranial magnetic stimulation (TMS) measures pre and post repetitive TMS (rTMS). The hypotheses were that fEMG would demonstrate excellent validity and agreement compared with sEMG.

**Materials and methods:**

In ten healthy volunteers, paired pulse and cortical silent period (CSP) TMS measures were collected before and after 1200 pulses of 1Hz rTMS to the motor cortex. Data were simultaneously recorded with sEMG and fEMG in the first dorsal interosseous. Concurrent validity (r and rho) and agreement (Tukey mean-difference) were calculated.

**Results:**

fEMG quantified corticospinal excitability with good to excellent validity compared to sEMG data at both pretest (r = 0.77–0.97) and posttest (r = 0.83–0.92). Pairwise comparisons indicated no difference between sEMG and fEMG for all outcomes; however, Tukey mean-difference plots display increased variance and questionable agreement for paired pulse outcomes. CSP displayed the highest estimates of validity and agreement. Paired pulse MEP responses recorded with fEMG displayed reduced validity, agreement and less sensitivity to changes in MEP amplitude compared to sEMG. Change scores following rTMS were not significantly different between sEMG and fEMG.

**Conclusion:**

fEMG electrodes are a valid means to measure CSP and paired pulse MEP responses. CSP displays the highest validity estimates, while caution is warranted when assessing paired pulse responses with fEMG. Corticospinal excitability and neuromodulatory aftereffects from rTMS may be assessed using fEMG.

## Introduction

Corticospinal excitability measured with transcranial magnetic stimulation (TMS) is nearly universally assessed via electromyography (EMG) electrodes placed on the skin’s surface (sEMG)[1]. sEMG is well suited to measure TMS responses because it captures the summation of activated motor units from corticomotor stimulation[2], resulting in a smooth motor evoked potential (MEP) trace that can be easily quantified by its amplitude. Dependence exclusively on sEMG, however, greatly limits the number of muscles and their corresponding cortical representation areas that can be investigated with TMS. Small, intrinsic, or deep muscles such as those used in vocalization, cervical and pelvic floor musculature have been studied with fine wire indwelling electrodes to determine the latency of activation from TMS[3–7]; however, these muscles remain virtually unexplored with conventional single or paired pulse intracortical excitability measures. No study has reported on the validity and agreement of multiunit intramuscular EMG to quantify corticospinal excitability measures compared to sEMG. Assessing corticospinal excitability in muscles inaccessible to sEMG will allow for a more comprehensive understanding of neurological disorders that affect intrinsic and deep muscles.

Intramuscular or indwelling fine wire electrodes (fEMG) capture activity from a small population of single motor units (SMUs) near to the electrode. Use of fEMG with TMS has been implemented to assess coil orientation effects on the latency of evoked responses[3,8], recruitment order of SMUs to increasing stimulation intensity[9], and hemispheric innervation to intrinsic musculature[6,10], while none report on the MEP amplitude or amplitude modulation in response to paired pulse stimuli. The advantage of using fEMG is that it may decrease cross-talk from nearby muscles[11], assess discharge properties of motor neurons originating in the spinal cord[12] and that it can capture low threshold and small motor units overlooked by sEMG[13]. In addition, actions that displace the skin such as pronation and supination hinder the use of sEMG while fEMG may provide a viable alternative method.

There is no literature reporting on the validity and agreement of multiunit fEMG to sEMG to quantify common corticospinal measures such as CSP and paired pulse tests. Further, it is unknown if corticospinal excitability measures using fEMG are sensitive to neuromodulation such as repetitive transcranial magnetic stimulation (rTMS). Therefore, before intramuscular targets can be used to measure corticospinal excitability or capture the effects of non-invasive brain stimulation, the validity of fEMG must be assessed against the commonly used sEMG method. With this purpose, we investigated TMS testing outcomes using simultaneous recording of fEMG and sEMG, before and after a session of inhibitory neuromodulation with rTMS.

## Materials and methods

### Participants

Eleven healthy, right-handed adults ages 24–35 (Mean ± SD: 25.6±3.2) were recruited and ten (4 male) completed the study. One subject failed to complete the experiment due to an equipment failure and could not return to the testing site. This study was approved by the University of Minnesota Institutional Review Board and Clinical Translational Science Institute and conformed to the ethical standards of the institutional research committee and with the 1964 Helsinki declaration. Written informed consent was obtained from all individual participants included in the study.

### Experimental design

A pretest/posttest design was used to compare responses both before and after one session of inhibitory (1Hz) rTMS. The pre and posttest assessment consisted of primary motor cortex excitability testing similar to a previously established protocol [14–16].

### Electromyography and transcranial magnetic stimulation

**Fig 1:**
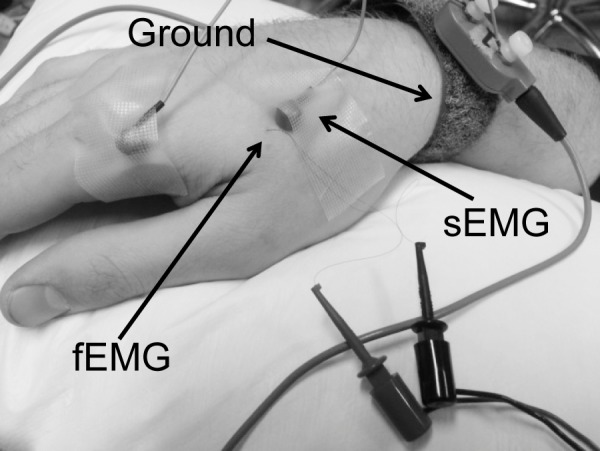
Electrode and ground placement. sEMG was placed in a belly-tendon montage while fEMG was inserted near the active surface electrode. Both sets of electrodes were pre-amplified with two custom made amplifiers. The ground was wrapped around the wrist. sEMG = surface electromyography; fEMG = fine wire electromyography.

Corticospinal excitability was assessed using both sEMG and fEMG simultaneously collected from the right first dorsal interosseous using a 70-mm figure-of-eight coil connected to a Magstim 200 stimulator (The Magstim Company Ltd, Carmarthenshire, UK). sEMG was recorded with a pair of bi-polar silver-silver chloride electrodes (EL254, BIOPAC System Inc., Aero Camino Goleta, CA). The active electrode was placed on the muscle belly and the reference on the metacarpophalangeal joint (Fig 1). fEMG activity was recorded with paired hook-wire electrodes (30mm x 27ga; Natus® Medical Inc., San Carlos, CA) inserted into the muscle near the active recording sEMG electrode. EMG signals were amplified by two bipolar EMG amplifiers (Y03-2, Motion Lab Systems, Inc., Baton Rouge, LA) with a gain of x300 and band-pass filter (20~2000Hz), then digitized by an analog-to-digital convertor (NI 9234, National Instruments, Austin, TX) with a 24-bit resolution at a sampling rate of 6.4 kHz. The ground electrode (TD-431, Discount Disposables, St. Albans, VT) was wrapped around the right wrist (Fig 1).

Subjects were seated in a comfortable, semi-reclined chair during testing. Real time EMG signals were displayed on a screen to insure relaxation of the target muscle. The coil was positioned with the handle directed posterolaterally 45° to the mid-sagittal line of the head over the approximate location of the optimal site to elicit an MEP, which is referred to as the hotspot. To determine the hotspot, single-pulse magnetic stimuli were delivered until a MEP was elicited and then the intensity of stimulation decreased to locate the site in the primary motor cortex with the lowest threshold muscle response. Resting motor threshold (RMT) was established similar to a previously established protocol[14–16]. The RMT was defined as the lowest intensity required to generate an MEP amplitude greater than 50μV three out of five trials[17] in the sEMG electrode. The 1 mV threshold was defined as the minimum intensity required to elicit an MEP amplitude of 1 mV in the sEMG electrode at least three out of five trials at the previously determined hotspot. Excitability measures consisted of single and paired pulse testing: CSP, short interval intracortical stimulation (SICI), and intracortical facilitation (ICF) and collected according to previously described methods[15,16]. For CSP testing, the participants contracted their right index finger against a barrier and the rectified average (using a 30ms window) EMG intensity was displayed in real time on a screen placed in front of the participant. Three trials of the participant’s maximum voluntary isometric contraction were recorded and 20% of the peak EMG intensity was used as a target line. During the test, participants performed an isometric contraction of the first dorsal interosseous and maintained the EMG intensity to the target line until instructed to relax. Ten trials of CSP were collected using 120% of RMT for each subject. SICI and ICF testing consisted of a subthreshold (80% RMT) conditioning stimulus and a suprathreshold testing stimulus (1mV threshold). SICI and ICF measurements had an interstimulus interval of 3ms and 13ms respectively. A total of 30 randomized trials were collected consisting of ten trials each of conditioned SICI and ICF responses as well as ten unconditioned single pulse responses at 1mV threshold. Testing of excitability measures occurred before and immediately after 1200 pulses of 1Hz rTMS at 90% of RMT delivered to the hotspot of the primary motor cortex hand area with a Magstim Rapid^2^ (The Magstim Company Ltd, Carmarthenshire, UK)[16]. Cortical stimulation location was spatially tracked throughout testing and rTMS delivery (BrainSight, Rogue Research Inc. Quebec, Canada) to insure consistent coil placement.

### Data processing

Peak-to-peak amplitude of each fEMG and sEMG trace was calculated for single and paired pulse responses for the SICI and ICF tests. The mean value of the 10 single pulses was calculated within each subject and each SICI and ICF value was divided by the mean single pulse value for pretest and posttest respectively. For CSP, the EMG data were first rectified, and then a 10ms moving standard deviation (SD) calculation was applied to the data. The onset of the CSP was the time point of the delivery of the stimulus. The average of the moving SD of the pre-stimulus data (-100ms to -5ms) was used as a threshold to determine the offset of the CSP. Offset was defined as the point that the moving SD value returned to the pre-stimulus level[18]. To compare changes in response after rTMS, a normalized individual change score for SICI, ICF and CSP were calculated using a linear transformation as: Normalized change score $=(Y-{\bar{X}}_{pre})/{\bar{X}}_{pre}$ Where, *Y* is the individual SICI, ICF or CSP values of the posttest; ${\bar{X}}_{pre}$ is the mean value of the individual SICI, ICF or CSP values of the pretest[16]. No outliers were removed from the data.

### Concurrent validity and agreement

A normality test (Kolmogorov-Smirnov) was conducted on all measures with the significance set at 0.05. To determine concurrent validity between fEMG and sEMG Pearson’s (r) or Spearman’s (rho) correlation coefficient was calculated, as appropriate for normal and non-normal distributions respectively. Correlation coefficient interpretation was based on general guidelines suggested by Portney and Watkins (2009) for both Pearson’s and Spearman’s coefficients: good to excellent relationship if r >0.75, moderate to good if r = 0.50 to 0.75, and fair if r = 0.25 to 0.50, and little to no relationship if r = 0.00 to 0.25[19]. To assess agreement between methods of EMG recording, individual trials were tested by paired Wilcoxon Signed Rank test (due to non-normally distribution as tested by Shapiro-Wilk W test). Individual trials were also visually examined by Tukey Mean-Difference plots, also known as Bland-Altman plots [20]. Graphical data was plotted with 95% CI limits of agreement to visualize differences between methods and 95% CI of the mean difference to assess systemic differences between methods[20]. Average values of each participant were tested by Tukey mean difference matched pair comparison (data were normally distributed according to Shapiro-Wilk W test). The significance level was set at p = 0.05. To compare sEMG and fEMG at each time point (before or after rTMS), all trials were included. Normalized change scores between fEMG and sEMG were compared with student’s t-tests using significance set at 0.05. Data were analyzed using SPSS (IBM Corp. Released 2011. IBM SPSS Statistics for Windows, Version 20.0. Armonk, NY: IBM Corporation).

## Results

**Fig 2:**
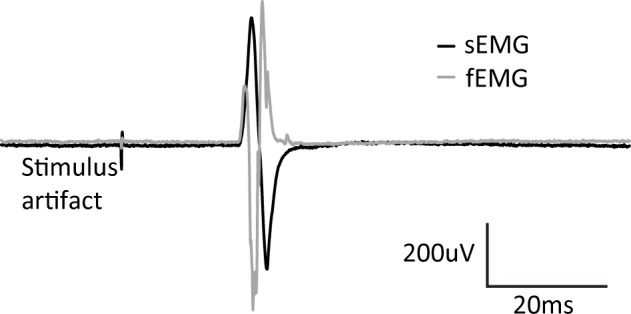
Simultaneous surface and fine-wire EMG trace. Example of a single pulse motor evoked potential response in a single subject.

Three of the ten subjects reported mild and short duration headaches following the testing session. No other adverse events were found. fEMG traces contained multiunit data sufficient to produce similar waveform to sEMG (Fig 2).

Table 1 lists validity estimates for all outcomes at pretest and posttest. Only CSP data at posttest was normally distributed and thus Pearson’s r was used to assess validity. SICI, ICF and CSP data at pretest was non-normally distributed and Spearman’s rho was calculated. CSP displayed excellent validity at pretest (rho = 0.97) and posttest (r = 0.92). ICF displayed excellent validity at pretest (rho = 0.83) and posttest (rho = 0.89). SICI was found to have excellent validity at pretest (rho = 0.77) and posttest (rho = 0.83).

**Table 1:** Validity at Pretest and Posttest.

Measure	Session	sEMG Mean±SD	fEMG Mean±SD	R
SICI	Pre	0.32±0.5	0.27±0.4	0.77
	Post	0.27±0.4	0.25±0.4	0.83
ICF	Pre	1.37±1.0	1.38±1.3	0.83
	Post	1.29±0.8	1.27±0.9	0.89
CSP	Pre	149.3±40.4	147.3±40.3	0.97
	Post	161.8±33.3	161.4±33.8	0.92

SICI = short interval intracortical inhibition; ICF = intracortical facilitation; CSP = cortical silent period; R = correlation coefficient.

Wilcoxon Signed Rank paired test on individual trials indicated no difference between EMG methods for CSP at pretest (p = 0.33) or posttest (p = 0.63) and no difference for ICF at pretest (p = 0.36) or posttest (p = 0.18). There was a significant difference between EMG methods for SICI at pretest (p = 0.04) but not at posttest (p = 0.15). Tukey’s mean difference matched pair comparisons indicated no difference between EMG methods in SICI at pretest (t = -0.4746, p = 0.65), SICI at posttest (t = -8464, p = 0.42), ICF at pretest (t = -0.0779, p = 0.94), ICF at posttest (t = -0.7019, p = 0.50), CSP at pretest (t = -1.0883, p = 0.31) and CSP at posttest (-0.2138, p = 0.84).

**Fig 3:**
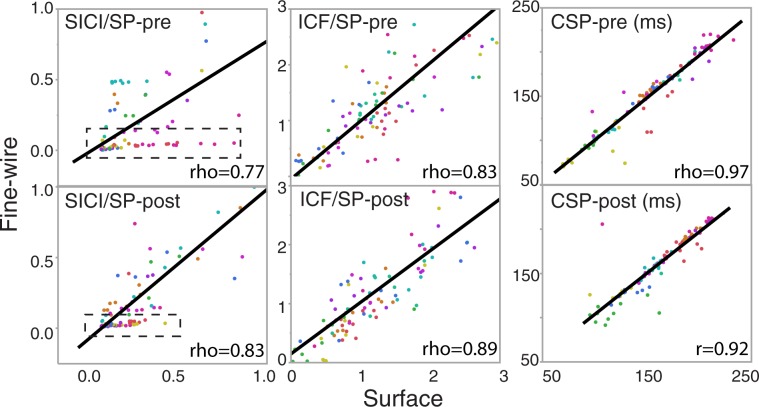
The relationship between sEMG and fEMG. Pretest data (top row) and posttest (bottom row). Data from 10 subjects, 10 trials/session/person, total of 100 data point in each plot. Note, the boxes on SICI graphs: fEMG responses cluster near 0 (y-distribution) while surface EMG response is more varied (x-distribution). Each color represents a single subject.

**Fig 4:**
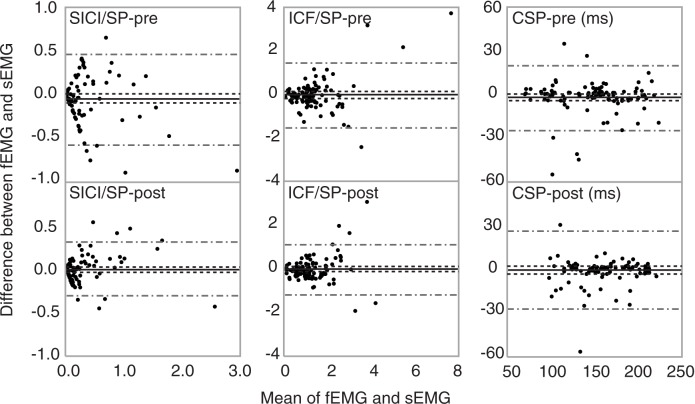
Agreement between sEMG and fEMG. Pretest data (top row) and posttest data (bottom row). Data from 10 subjects, 10 trials/session/person, total of 100 data point in each plot, 95% CI of limits of agreement (wide grey lines) and 95% CI of the mean (black lines).

Visual analysis of the relationship between sEMG and fEMG at pretest and posttest revealed a large number of SICI estimates by fEMG clustered near to zero, while sEMG estimates demonstrated a larger range of values (Fig 3, boxes surround data points). Lack of fEMG to detect SICI amplitude variance suggests that fEMG may be less sensitive than sEMG in SICI measurements. Similarly, Tukey mean difference plots also display increased variance (y-axis) with increased mean estimates (x-axis) for SICI and ICF (Fig 4).

Following rTMS, normalized change scores were not statistically different between electrode types for all outcomes. There was a tendency for fEMG to underestimate change scores of paired pulse responses as evident in the normalized change scores (Table 2). fEMG reflected approximately 60% of the sEMG value. These results indicate that fEMG detected less change in intracortical excitability using paired pulse tests following rTMS than sEMG.

**Table 2:** Normalized Group Change Scores.

Measure	Electrode	Mean ± SD	P-Value	t
SICI	sEMG	0.485±1.10	0.35	0.995
	fEMG	0.268±1.02		
ICF	sEMG	0.136±0.69	0.68	0.421
	fEMG	0.084±0.60		
CSP	sEMG	0.118±0.19	0.65	-0.473
	fEMG	0.130±0.21		

SICI = short interval intracortical inhibition; ICF = intracortical facilitation; CSP = cortical silent period; sEMG = surface electromyography; fEMG = fine-wire electromyography; SD = standard deviation.

## Discussion

Our results indicate that fine wire electrodes are a valid means of collecting the EMG signal for TMS measures including SICI, ICF and CSP. Among these measures, CSP displayed the highest validity and agreement while paired pulse responses were found to have excellent validity despite variable agreement when compared to sEMG. SICI data should be interpreted with caution when using fEMG, as results may be underestimated.

Previous studies in the first dorsal interosseous have shown that fEMG data are sensitive to the conditioning effects of paired inter- and intra- hemispheric TMS using peri-stimulus time histograms[21,22]. Our results support this finding with results of good to excellent validity of fEMG to quantify SICI and ICF MEP responses. Additionally, all Tukey mean difference plots displayed the line of equality within the 95% CI of mean, indicating low bias and lack of systemic difference between methods for all measures[20]. However, our SICI data as measured by fEMG appeared to have questionable agreement at pretest (Fig 4) and be less sensitive to changes in SICI MEP amplitude compared to sEMG (Fig 3).

The clustering effect of data near to zero in correlation graphs, and the lower estimates of validity and graphical agreement for ICF and SICI in fEMG compared to sEMG may be the result of a limited pool of motor units that summate on the indwelling electrode. A small sample of motor units within the recording area may limit the ability for the fEMG trace to vary in amplitude across trials, unlike sEMG that assess a larger sample of motor units. Since ICF and SICI are calculated based on a ratio of MEP amplitude, this finding should be carefully considered in future work. CSP duration would not be influenced as greatly by this, as it is quantified using the timing of EMG activation, not the amplitude of the MEP. SICI responses may be more susceptible to a floor effect, as SICI reflects the ratio of the paired pulse inhibited MEP amplitude to the single pulse MEP amplitude. If only few motor units are within the recording area, a paired pulse SICI test may result in full suppression of all motor units and hence a very small MEP amplitude or no response could be recorded in the fEMG trace. Thus, the outlined SICI data points near zero fEMG (Fig 3) may reflect missing or undetected SICI responses using the protocol here. One technique to better quantify SICI may be to use a pre-established level of stimulator output for the testing stimulus. In this technique, the stimulus should produce 50% of the maximal MEP amplitude for the testing stimulus so that it may be sufficiently facilitated or inhibited by the conditioning stimulus[23].

Our results support the use of CSP to assess the effects of rTMS on corticospinal excitability, specifically GABA_B_ medicated intracortical inhibition, as CSP displayed the highest levels of validity and agreement for both fEMG and sEMG. However, it has been reported that SMU based analyses, such as a peristimulus frequencygram, estimate a longer CSP compared to probability based analyses and standard sEMG[24]. Todd and colleagues suggest that the duration of inhibitory events at motor neuron pools may contribute to these differential results based on the method of EMG analysis[24]. Accordingly, intramuscular recordings largely reflect activity in low threshold and small SMUs, generating additional information that may be overlooked in sEMG[13]. Yet, using the CSP analysis technique here there was no difference between fEMG and sEMG. Our method provides a more time efficient and feasible protocol to assess corticospinal excitability using multiunit fEMG data compared to SMU based methods.

There are limitations to this study. One limitation is that the number of SMUs recorded in each subject were not assessed. If too few SMUs lie within the recording area, there may be insufficient summation of motor units for amplitude modulation in response to paired pulse protocols. This may be one reason fEMG is less sensitive to changes in SICI, as too few SMUs would not allow amplitude modulation of the EMG trace. It is unknown how many MUs are required to generate reliable measures of corticospinal excitability. Additionally, the RMT for the TMS intensity was determined using the sEMG traces, which may not translate to the same threshold with fEMG. This concern is lessened because threshold values are nearly the same (<5% of maximal stimulator output) using sEMG based on amplitude and fEMG data based on SMU firing probability[22]. Lastly, this study did not evaluate an active MEP response. This study was only intended to evaluate commonly used TMS outcomes and thus tests such as CSP (which are collected in an active muscle), only evaluated the duration of the silent period and not the MEP amplitude. This would be valuable information for future studies to assess.

## Conclusion

Our results indicate that CSP and paired pulse outcomes display excellent validity while agreement varied between testing outcomes. This methodology may be used in other venues of neurophysiology research that seeks to perform comprehensive TMS testing and delineate neuromodulatory effects in musculature that is inaccessible to sEMG. Future applications may aim to enhance neuromotor control to pelvic floor muscles, the diaphragm or laryngeal musculature.

## Supporting information

S1 Appendix Pretest and posttest data. Data for outcomes at both pretest and posttest are provided for sEMG and fEMG. Tabs include separate outcomes: Short-interval intracortical inhibition (SICI), intracortical facilitation (ICF), cortical silent period (CSP).
(XLSX)
